# Antioxidant Activity and Oxidative Damage Associated with Seeding Surgery for Pearl Culture in the Winged Pearl Oyster *Pteria sterna*

**DOI:** 10.3390/antiox13060723

**Published:** 2024-06-14

**Authors:** Andrés Granados-Amores, Ángel I. Campa-Córdova, Héctor Acosta-Salmón, Carlos Angulo, Tania Zenteno-Savín, Carmen Rodríguez-Jaramillo, Pedro E. Saucedo

**Affiliations:** 1 Centro de Investigaciones Biológicas del Noroeste (CIBNOR), La Paz 23096, Baja California Sur, Mexico; a.granados@uabcs.mx (A.G.-A.); angcamp04@cibnor.mx (Á.I.C.-C.); hacostas@cibnor.mx (H.A.-S.); eangulo@cibnor.mx (C.A.); tzenteno04@cibnor.mx (T.Z.-S.); jaramilo04@cibnor.mx (C.R.-J.); 2 Departamento Académico de Ingeniería en Pesquerías at Pichilingue, Universidad Autónoma de Baja California Sur at Pichilingue, La Paz 23000, Baja California Sur, Mexico

**Keywords:** mantle tissue, aquaculture, pearl culture, autograft, allograft, antioxidant activity, oxidative damage

## Abstract

To evaluate the antioxidant activity and oxidative damage by relaxing, wounding, and seeding of a saibo of different origin on *Pteria sterna* hosts, five oyster treatments were included: (1) relaxed (REL) but neither wounded nor seeded; (2) relaxed and wounded (WOU) but not seeded; (3) relaxed, wounded, and seeded with an allograft (ALL); (4) relaxed, wounded, and seeded with an autograft (AUT); and (5) unrelaxed, unwounded, and unseeded as control (CTR). Superoxide dismutase (SOD), catalase (CAT), glutathione peroxidase (GPx), and thiobarbituric acid (TBARS) activity were quantified between 3 and 24 h post-seeding. Compared to the CTR oysters, which did not suffer oxidative stress, SOD activity significantly decreased in the gonad and digestive gland in all treatments and decreased in mantle tissue in AUT oysters; this indicates that the entire process of preparing oysters for pearl culture (relaxing, wounding, and seeding) generates oxidative stress in the host. CAT was not a sensitive enzyme for measuring the short-term response of oysters to the wounding–seeding processes but rather a more prolonged or chronic stress. Similar to SOD, the lowest GPx and TBARS activity in seeded oysters evidenced their susceptibility to oxidative stress and damage, particularly in the WOU treatment. Evidence from this study indicates that SOD is a more sensitive enzyme for measuring the short-term response of the host oyster to the wounding and seeding of a saibo. It is also clear that the host undergoes stress at all stages of the pearl culture process, mostly during gonad wounding and regardless of the origin of saibo.

## 1. Introduction

In commercial pearl farming, the seeding of a spherical bead and a piece of mantle tissue (saibo) is an invasive surgical procedure that is not only stressful to the host mollusk but may result in its death [1,2]. In pearl oysters of the genus *Pinctada*, the usual procedure for seeding the host is to obtain the saibo from a donor of the same species (allograft), but under particular conditions, it can also be obtained from a donor of a different species (xenograft) or from the same oyster that is seeded (autograft) [3,4]. An allograft makes it possible to produce commercial lines of pearls with species-specific optical qualities of the nacre, such as color and luster [1]. With an autograft, no separate donor oyster is needed to prepare the saibo, but its seeding may require greater mobilization of energy from the host oyster to repair a double wound: in the mantle to remove the saibo and in the gonad where it is seeded [5,6]. Finally, xenografting is another strategy that appears to favor survival and bead retention in the host oyster, as well as some key optical properties of the nacre (thickness and weight) and resulting pearls (color, brightness) [7,8]. In all cases, the host response to seeding is species-specific and depends on complex immune and antioxidant reactions that have been little studied in pearl oysters [1,2,3,4,7,8,9].

In bivalve mollusks, the control of oxidative stress is carried out by different types of effector agents of the antioxidant and innate immune system, including circulating hemocytes; chaperones or heat shock proteins; and enzymes such as superoxide dismutase (SOD), catalase (CAT), glutathione peroxidase (GPx), and glutathione reductase (GRx), among others [10,11,12]. The antioxidant system in particular protects cells against a wide variety of acute and chronic stressors (xenobiotics) and counteracts the negative effects of reactive oxygen species (ROS) [13,14]. A temporary or permanent unbalance between prooxidants and antioxidant defenses is mostly attributed to exogenous factors (i.e., heavy metals, hydrocarbons, biotoxins, infectious diseases, and environmental changes), as well as to physiological deficiencies in the animal [15,16,17,18]. For this reason, antioxidant enzymes (SOD, CAT, GPx, and GRx), together with the production of substances such as thiobarbituric acid (TBARS), have been considered reliable indicators of oxidative stress and oxidative damage in marine and freshwater species [11,13]. In pearl oysters in particular, the role of the antioxidant system has only been correlated with the presence of hydrocarbons and heavy metals [19,20,21], but its regulation due to seeding surgery for pearl culture is unknown in these organisms.

The winged pearl oyster *Pteria sterna* (Gould, 1851) is the only species of this genus currently used for culturing bead-seeded pearls in the eastern Pacific, where it supports more than 95% of the commercial pearl production [2]. These pearls are recognized by their dominant light gray to jet black colors and multicolor green, violet, blue, and golden overtones, which are in high demand and clearly differentiated from pearls produced by all *Pinctada* species [22]. However, as the average production of *P. sterna* pearls is relatively low (20–35%) due to high mortality and bead rejection rates [2], this study evaluated the effects of wounding and seeding of an autograft and allograft on the host antioxidant activity and oxidative damage, testing two hypotheses: (1) a saibo seeded in the soft body of the host is a foreign body or xenobiotic that triggers a battery of physiological responses, and (2) healing from the seeding of an autograft (which involves a double wound in the mantle and gonad) is more stressful for the host than healing from the seeding of an allograft (only involving a single wound in the gonad).

## 2. Materials and Methods

### 2.1. Experimental Design

This study used 150 *P. sterna* adults (110 mm ± 5 mm mean shell height) that were collected as juveniles at the pearl farm Perlas de La Paz, in La Paz, Baja California Sur, Mexico. These oysters remained under culture conditions for two years in plastic culture baskets hung from 50 m longlines at a depth of 10 m before being used for the current study. Once extracted, they were cleaned, measured (0.1 mm), wet-weighed (0.1 g), and divided into four experimental treatments of 10 oysters each to evaluate the effect of seeding surgery and saibo origin. Each treatment was managed in triplicate (n = 30). The treatments included (1) oysters relaxed (REL) but neither wounded in the gonad nor seeded; (2) oysters relaxed and wounded (WOU) in the gonad but not seeded; (3) oysters relaxed, wounded in the gonad, and seeded with an allograft (ALL); and (4) oysters relaxed, wounded in the gonad, and seeded with an autograft (AUT). A control group (CTR) of unrelaxed, unwounded, and unseeded oysters was also included (n = 30).

**Figure 1:**
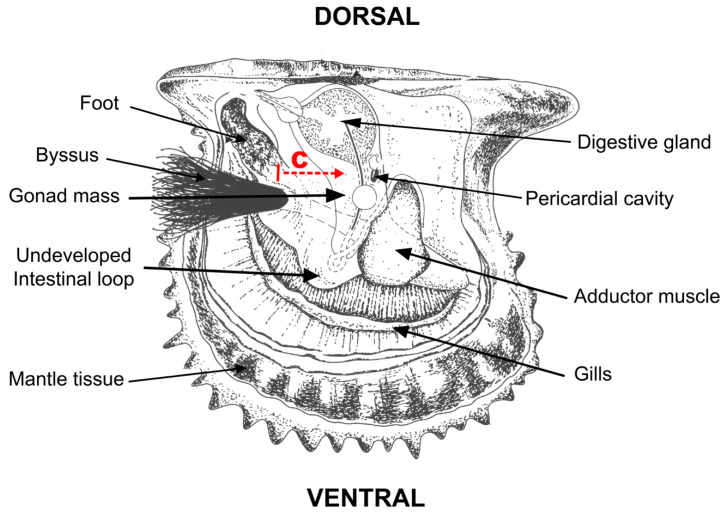
Internal anatomy of the winged pearl oyster *Pteria sterna*, showing the body area (base of the foot) where the incision is made (solid red line) and the direction of the canal (c) opened toward the most dorsal part of the gonad (dashed red line) during seeding surgery. The diagram also shows the lack of a developed intestinal loop in this pearl oyster species for seeding the bead and saibo, unlike members of the genus *Pinctada*.

Oysters from the AUT and ALL treatments were seeded following the protocol defined for *P. sterna* [2,23]. As this species does not have an intestinal loop, or if present it is too small, the incision was made at the base of the foot, and the canal was opened toward the most dorsal area of the gonad, where the bead and saibo were inserted (Figure 1). In this case, the oysters received only the saibo—the organic tissue that causes irritation and triggers a physiological response—but not the inert bead. The surgery was always performed by the same skilled technician. Except for the CTR oysters that remained in raw seawater at the same conditions as in the wild, those from the other treatments were previously relaxed with 2.5 mL L^−1^ propylene phenoxetol for 45 min [24]. In terms of logistics, each experimental treatment was processed on a separate day.

Once seeded, the oysters were transferred to post-operative care units to identify and discard any dead oysters and determine individual and total survival (%) at each treatment. Then, two oysters per replicate (six oysters per treatment) were collected at 3 h, 6 h, 9 h, 12 h, and 24 h post-seeding to excise tissue samples (gonad, digestive gland, and mantle tissue), which were preserved at –80 °C. These samples, together with those obtained at the start of the trials before seeding the oysters (t0), were used to determine the activity of antioxidant enzymes (SOD, CAT, and GPx) and oxidative damage (TBARS), following the standard protocols described below.

### 2.2. Analysis of Antioxidant Activity

Preserved tissue subsamples (100 mg) were firstly lyophilized and homogenized in a cold phosphate-buffered solution (50 mM, pH 7.5) and a phenyl–methyl–sulphonyl–fluoride solution (0.5 mM). They were then centrifuged (2124× *g*; 4 °C), and the supernatant was carefully recovered to prepare tissue homogenates. Previously, the concentration of total proteins per tissue and sampling time was determined using a commercial colorimetric reagent (#B6916, Sigma-Aldrich, St. Louis, MO, USA) and bovine serum albumin (#9048-46-8, Sigma-Aldrich) as the standard [25]. Absorbance was measured in triplicate in a microplate reader at 620 nm, and the results were expressed as mg mL^−1^ protein.

#### 2.2.1. Superoxide Dismutase (SOD)

The method of Suzuki [26] was used to determine the activity of SOD enzyme. Tissue homogenate subsamples (25 µL) were mixed with 1.5 mL sodium carbonate (50 mM) as a buffer, xanthine (0.1 mM), nitroblue tetrazolium (0.025 mM, EDTA 0.1 mM), and xanthine oxidase solution (1 U XO, 1 mL ammonium sulfate 2 M). Each sample was analyzed in triplicate, and absorbance was recorded at 560 nm every 30 sec for 5 min. A unit of activity of SOD was defined as the amount of enzyme that inhibited 50% of the reaction of O_2_^−^ with nitroblue tetrazolium. The results were expressed as U SOD mg^−1^ protein.

#### 2.2.2. Catalase (CAT)

To determine the activity of CAT, the procedures of Aebi [27] were adopted. Tissue homogenate subsamples (10 µL) were placed in a quartz cell with 1.5 mL working solution (0.1 M phosphate-buffered solution; pH 7.0) and 20 nM hydrogen peroxide. The reading was carried out in triplicate at 240 nm every 15 s for 3 min. A unit of activity of CAT was defined as the amount of enzyme that catalyzed the reduction of 1 µmol H_2_O_2_ per min. The results were expressed as U CAT mg^−1^ protein.

#### 2.2.3. Glutathione Peroxidase (GPx)

The activity of GPx was determined with the protocol of Flohé and Günzler [28]. Three pattern solutions were prepared: (1) a mixture of phosphate buffer (500 mM; pH 7.2), EDTA (50 mM), and acid sodium solution (20 nM); (2) glutathione reductase (7.5 U mL^−1^), phosphorylated-reduced nicotianamine and adenine (1.5 mM NADPH), and reduced glutathione (250 mM); and (3) deionized and oxygenated water (10 mM). Deionized water (25 µL) was mixed with solutions 1 and 2, incubated at 37 °C for 10 min, read in a microplate at 340 nm, and analyzed using the NADPH extinction coefficient (0.00373 µM^−1^). The results were expressed as U mg^−1^ protein, considering one unit of activity of GPx as the amount of enzyme needed to oxidize 1 nmol NADPH to NADP+ per min.

### 2.3. Analysis of Oxidative Damage

The procedure of Ohkawa [29] was adopted to quantify the substances reactive to thiobarbituric acid (TBARS). Tissue homogenate subsamples (250 µL) were incubated in distilled water at 37 °C for 15 min and cooled to 4 °C for 15 min. To stop the reaction, a mixture of 20% trichloroacetic acid and 1.0 mol mL^−1^ of hydrochloric acid was added. Samples were stirred in a water bath at 90 °C, cooled again to 4 °C, and centrifuged at 2124× *g* for 10 min. The recovered supernatant was read in triplicate at 560 nm. The TBARS concentration was expressed as nmol TBARS mg^−1^ protein.

### 2.4. Statistical Analysis

Group normality was analyzed with the Kolmogorov–Smirnov test and confirmed with the Levene test for homogeneity of variances [30]. Given the lack of normal distribution and the small sample size per treatment at each sampling time (n = 6), a Kruskal–Wallis test was run to detect significant differences in the antioxidant (SOD, CAT, and GPx) and oxidant (TBARS) indicators between seeding treatments. The analyses were separately run for each body tissue (gonad, digestive gland, and mantle tissue) and at each sampling (3 h, 6 h, 9 h, 12 h, and 24 h). As needed, post hoc multiple-range comparisons with Tukey’s test (HSD) were included. All analyses were performed with Statistica 9.0 (TIBCO Software, Palo Alto, CA, USA) at a significance level set at *p* < 0.05 in all cases.

## 3. Results

All oysters in the CTR and REL treatments had 100% survival up to 24 h after seeding. The lowest survival at 24 h (93%) occurred in the ALL treatment. Significant differences in the activity of all antioxidant and oxidative damage enzymes were observed in most tissues of all experimental treatments.

### 3.1. Antioxidant Activity

#### 3.1.1. Superoxide Dismutase (SOD)

**Figure 2:**
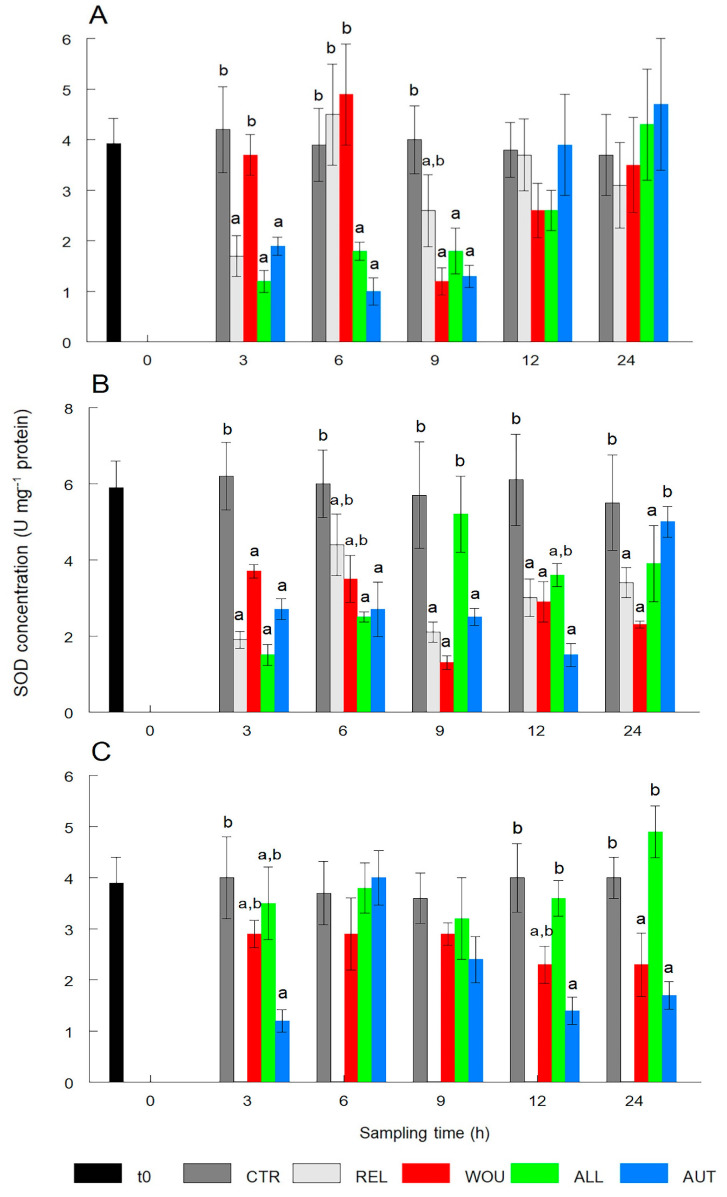
Temporal variations in superoxide dismutase (SOD) activity in the gonad (**A**), digestive gland (**B**), and mantle tissue (**C**) of the winged pearl oyster *Pteria sterna* subjected to different seeding treatments. Data include the mean ± standard error. Different superscript letters above the columns denote significant differences between treatments at *p* < 0.05. CTR = control group; REL = relaxed; WOU = wounded; ALL = allograft; AUT = autograft. Data from WOU treatment in mantle tissue (**C**) were missing and were not available for analysis.

With the exception of oysters in the WOU treatment, SOD content in the gonad significantly (*p* < 0.05) dropped after 3 to 9 h, especially in the AUT and ALL treatments, and returned to normal conditions after 12 and 24 h. Oysters in the WOU treatment only showed significantly (*p* < 0.05) lower levels of SOD 9 h after wounding. SOD levels in the gonad of CTR oysters remained within a narrow range and showed no significant (*p* > 0.05) differences between sampling times (Figure 2A).

Similarly, the SOD content in the digestive gland of oysters in all treatments significantly (*p* < 0.05) dropped after 3 h. SOD in all treatments remained significantly low and with some fluctuations, and only SOD values in the digestive gland of oysters in the AUT treatment returned to normal conditions after 24 h. SOD levels in the digestive gland of CTR oysters remained within a narrow range and showed no significant differences (*p* > 0.05) between sampling times (Figure 2B).

The SOD content in mantle tissue did not show a clear response to the treatments, and in the ALL treatment, values remained similar to the CTR with no significant (*p* < 0.05) variations throughout the experiment. The SOD content in the mantle tissue of AUT oysters showed two significant drops at 3 h and 12 to 24 h. SOD levels in the mantle of CTR oysters remained within a narrow range and showed no significant differences (*p* > 0.05) between sampling times (Figure 2C).

#### 3.1.2. Catalase (CAT)

**Figure 3:**
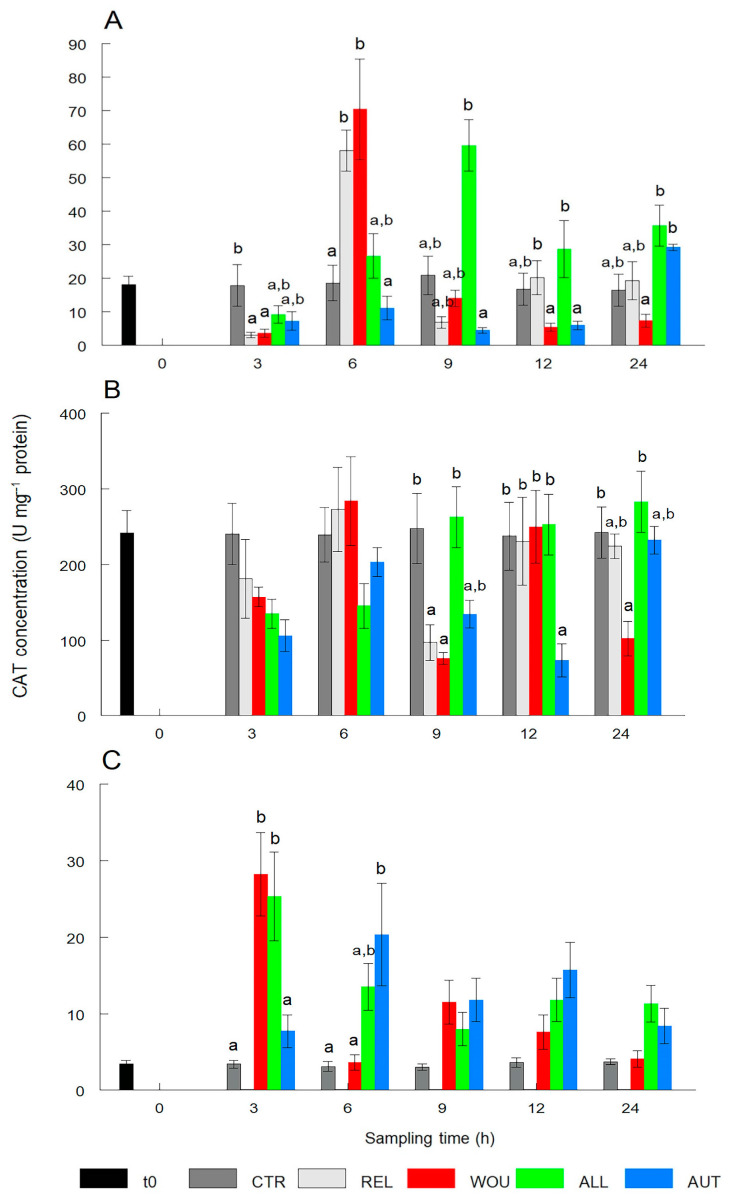
Temporal variations in catalase (CAT) activity in the gonad (**A**), digestive gland (**B**), and mantle tissue (**C**) of the winged pearl oyster *Pteria sterna* subjected to different seeding treatments. Data include the mean ± standard error. Different superscript letters above the columns denote significant differences between treatments at *p* < 0.05. CTR = control group; REL = relaxed; WOU = wounded; ALL = allograft; AUT = autograft. Data from WOU treatment in mantle tissue (**C**) were missing and were not available for analysis.

CAT in the gonad of oysters in the REL and WOU treatments significantly (*p* < 0.05) decreased after 3 h and then significantly (*p* < 0.05) increased almost three-fold after 6 h and returned to normal levels after 9 h. CAT in the gonad of oysters in AUT and ALL treatments remained similar to CTR oysters within a narrow range and showed no significant differences (*p* > 0.05) between sampling times (Figure 3A).

CAT activity in the digestive gland was relatively stable over time and was only significantly (*p* < 0.05) lower at 9 h after seeding in REL and WOU oysters. While CAT activity decreased significantly only in the AUT treatment at 12 h, significantly (*p* < 0.05) lower CAT values occurred at 24 h in oysters in the WOU treatment (Figure 3B).

In mantle tissue, CAT values significantly (*p* < 0.05) increased in WOU and ALL oysters at 3 h post-seeding, and in AUT oysters at 6 h. No significant differences (*p* > 0.05) in CAT activity were observed between treatments in mantle tissue from 9 to 24 h (Figure 3C).

#### 3.1.3. Glutathione Peroxidase (GPx)

**Figure 4:**
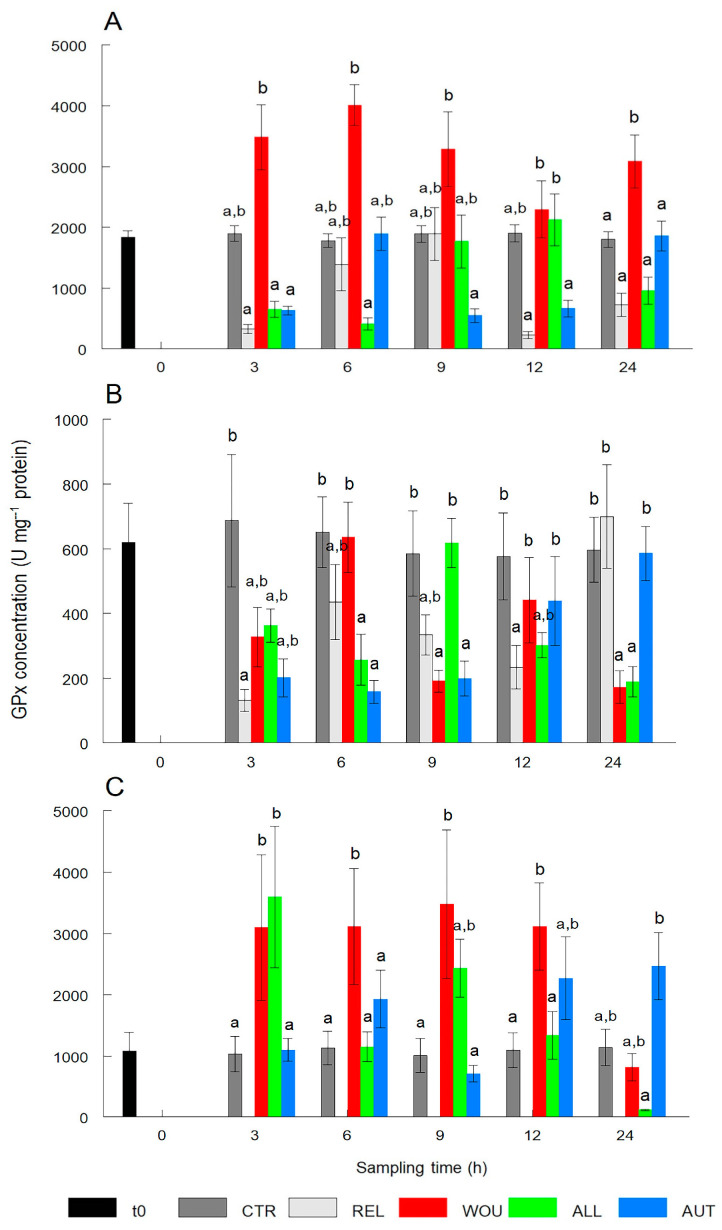
Temporal variations in glutathione peroxidase (GPx) activity in the gonad (**A**), digestive gland (**B**), and mantle tissue (**C**) of the winged pearl oyster *Pteria sterna* subjected to different seeding treatments. Data include the mean ± standard error. Different superscript letters above the columns denote significant differences between treatments at *p* < 0.05. CTR = control group; REL = relaxed; WOU = wounded; ALL = allograft; AUT = autograft. Data from WOU treatment in mantle tissue (**C**) were missing and were not available for analysis.

GPx content in the gonad did not significantly (*p* > 0.05) vary between treatments in most cases. Significant differences (*p* < 0.05) were only noticed between the GPx content in the gonad of oysters of different treatments but not between the CTR group. The GPx content in the gonad of CTR oysters remained within a narrow range and showed no significant differences between sampling times (Figure 4A).

The GPx activity in the digestive gland was erratic over time; it significantly (*p* < 0.05) decreased at 3 h only in REL oysters. Differences were significantly lower (*p* < 0.05) in the ALL and AUT oysters at 6 h and in the WOU and AUT oysters at 9 h. The GPx activity significantly (*p* < 0.05) decreased only in REL oysters at 12 h and in WOU and ALL oysters at 24 h after seeding (Figure 4B).

In mantle tissue, the GPx activity was constantly and significantly (*p* < 0.05) higher in the WOU treatment than in all other treatments between 3 h and 12 h and returned to normal levels at 24 h. GPx in the mantle of oysters in all other treatments was not significantly different (*p* > 0.05) from GPx in CTR oysters (Figure 4C).

### 3.2. Oxidative Damage

**Figure 5:**
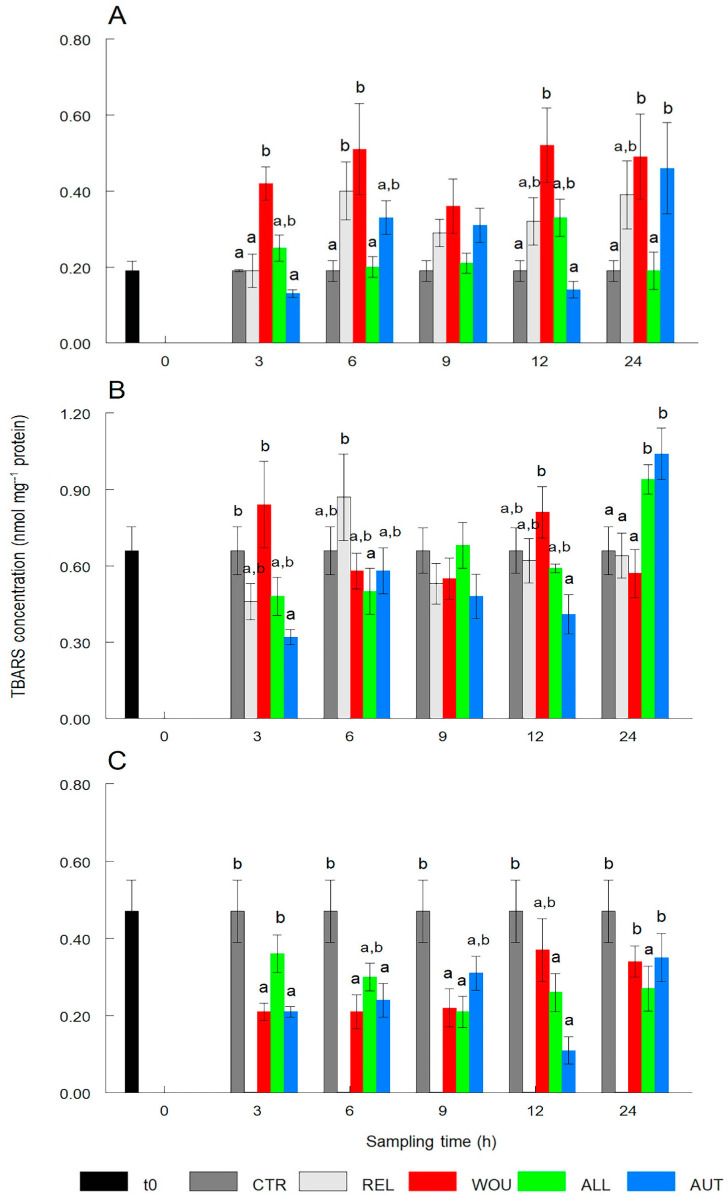
Temporal variations in the production of thiobarbituric acid (TBARS) in the gonad (**A**), digestive gland (**B**), and mantle tissue (**C**) of the winged pearl oyster *Pteria sterna* subjected to different seeding treatments. Data include the mean ± standard error. Different superscript letters above the columns denote significant differences between treatments at *p* < 0.05. CTR = control group; REL = relaxed; WOU = wounded; ALL = allograft; AUT = autograft. Data from WOU treatment in mantle tissue (**C**) were missing and were not available for analysis.

TBARS content significantly (*p* < 0.05) increased in the gonad of WOU oysters after 3 h and, with the exception of 9 h, remained significantly higher at all other sampling times and did not return to normal level after 24 h. REL oysters showed only significantly (*p* < 0.05) higher TBARS content after 6 h, while AUT oysters showed significant (*p* < 0.05) increases in TBARS concentration after 24 h (Figure 5A).

The TBARS content in the digestive gland remained without variations throughout sampling times, with the exception of oysters in the AUT treatment that showed significantly (*p* < 0.05) lower values after 3 h, and oysters in AUT and ALL treatments that showed a significant (*p* < 0.05) increase in the TBARS content after 24 h (Figure 5B).

The TBARS content in mantle tissue showed a significant (*p* < 0.05) decrease in oysters of most treatments throughout the sampling period. TBARS in the mantle of WOU and AUT oysters dropped significantly (*p* < 0.05) after 3 h and 6 h and returned to normal levels after 24 h. The TBARS content in ALL oysters dropped significantly (*p* < 0.05) after 9 h and did not return to normal levels after 24 h after seeding (Figure 5C).

## 4. Discussion

This study is the first to report antioxidant enzyme activity and oxidative damage in the winged pearl oyster *P. sterna* following allograft and autograft seeding for pearl farming. Assuming that seeding surgery is stressful for the host oyster [1,4,7], the hypothesis tested here was based on the fact that ROS are produced in living organisms during aerobic metabolism, but stress conditions that alter their production (wounding and seeding in this case) may result in oxidative stress (unbalance between antioxidant capacity and ROS production) and oxidative damage (i.e., lipid peroxidation) [12,13]. The evidence indicates that survival after seeding was not influenced by any experimental treatment (only 2 dead oysters out of 30 in the ALL treatment) and that stress response occurred in all oysters (REL, WOU, ALL, and AUT), which in comparison to CTR oysters, was reflected in significant differences in all indicators measured in most tissues and sampling times.

With the SOD enzyme, which converts anion superoxide (O_2_^−^) into hydrogen peroxide (H_2_O_2_), three patterns were clear. First, it was consistently and significantly lower in the gonad and digestive gland of oysters from all experimental treatments than in the CTR, confirming that SOD actively participated in both tissues after oysters were seeded, and particularly, that oysters that remained unrelaxed, unwounded, and unseeded did not undergo oxidative stress. Secondly, SOD activity was significantly decreased in the gonad of the ALL or AUT treatments from the first sampling, suggesting that the greatest stress load to the host occurred by the wound itself and subsequently by the seeding surgery. Third, the SOD activity in mantle tissue was also significantly lower in AUT oysters than in the ALL and CTR at most samplings, supporting the second hypothesis that the healing of a double wound (mantle and gonad) may be more stressful for the host oyster than the healing of a single wound (gonad). These results provide additional evidence for the possible involvement of specific tissue-resident cells in the antioxidant response of presumably stressed species [13], represented in this study by WOU, ALL, and AUT oysters.

In filter-feeder mollusks exposed to adverse environmental conditions (i.e., heavy metals, hydrocarbons, agricultural effluents, industrial wastewater, red tides, and biotoxins), variations in SOD activity have proved to be species- and stage-specific, but also depend on the type and duration of the condition [11,21]. In these cases, SOD has been used as a biomarker of water pollution in many marine and freshwater mollusks, including pearl oysters [16,18,19,20,21]. For instance, the SOD activity remained stable during short exposures to cooper concentrations in the Antarctic scallop *Adamussium colbecki* but significantly decreased during prolonged exposures to mercury [31]. Conversely, SOD levels showed a 1.8-fold increase in juvenile lion’s paw scallop *Nodipecten subnodosus* exposed to the toxic dinoflagellate *Prorocentrum lima*, compared to control scallops [32]. These results, together with those of the present study, indicate that SOD is a sensitive enzyme for assessing oxidative stress in *P. sterna* in response to the wounding and seeding of an autograft or allograft.

In contrast to the SOD activity, which affected all treatments, CAT patterns were mostly noticed in the REL and WOU treatments. Since oysters receiving an autograft or allograft were also relaxed and wounded, this situation suggests either of the following three options: (1) the seeding of a saibo through surgery somehow contributes to mitigating the antioxidant response of CAT; (2) the higher the concentration of H_2_O_2_ and other compounds, the more CAT can be inhibited; or (3) the response time measured in the study was short (24 h), and CAT was not a very sensitive enzyme for measuring the short-term response of oysters to the stress caused by the wounding–seeding process, but rather a more prolonged chronic stress; this has yet to be confirmed. Also, in *P. sterna* anesthetized for bead seeding, low CAT values were observed in tissues with large surface area (gills) exposed to substances such as eugenol and benzocaine, compared to the control [24]. Interestingly, in our study, CAT values in the digestive gland were 10 times higher than in the gonad and 20 times higher than in the mantle tissue in most treatments. Similarly, the CAT activity was also increased 10-fold in the digestive gland and gills of the mollusks *Unio pictorum* [33] and *Anadara inaequivalvis* [34], even when they were not exposed to stressful conditions. These findings appear to reflect the double role of the digestive gland in the storage and mobilization of energy reserves to sustain reproduction, as well as in the detoxification of cells from harmful substances through digestive processes [18,35].

The GPx enzyme, like all peroxidases, competes to some extent with CAT for H_2_O_2_ and protects lipid cell membranes from the harmful effects of some hydroperoxides [11,13]. Initially, this competition is consistent with the maximal activity of GPx in the gonad and mantle tissue and its minimal activity in the digestive gland, which showed an opposite pattern to that of CAT activity. Although GPx values did not vary much between treatments and CTR from 3 to 12 h post-seeding, a highly significant peak in the GPx activity occurred in WOU oysters in particular, both in the gonad and mantle tissue, highlighting the stressful effect of wounding. In line with this pattern, the TBARS content significantly peaked in the gonad of WOU oysters a

After seeding and dropped in the mantle tissue of oysters with an autograft. Together, these findings confirm that the greatest stress load and susceptibility to oxidative damage to the host oyster are firstly caused by wounding the gonad and subsequently by the seeding of the saibo, particularly an autograft; this last result somehow contradicts the test hypothesis. The REL treatment occupied an intermediate position in terms of the GPx and TBARS activity. In relation to the use of a saibo for seeding surgery and pearl culture, some authors [5,6] have reported that the pearl oysters *Pinctada fucata*, *Pinctada margaritifera*, and *Pinctada maxima* can fully regenerate the excised mantle in 30–40 days with no signs of cellular damage or death. In other bivalve mollusks such as the river mussel *Unio tumidus* and the pearl oyster *P. fucata*, GPx was reported as one of the main antioxidant enzymes that prevent ROS overproduction and lipid peroxidation due to water pollution by sewage, industrial waste, and high copper concentrations [20,36].

In light of the results of this study, it appears that the hypothesis stating that seeding *P. sterna* with an autograft would cause less oxidative stress and oxidative damage than seeding with an allograft was not fully confirmed. Rather, the data suggest that the antioxidant response by the host is influenced more by the wound itself (gonad or mantle) than by the origin of the saibo. Based on these results, new hypotheses for future research with this pearl oyster species can be drawn to determine whether seeded oysters can (1) suppress antioxidant response and ROS overproduction; (2) activate nonenzymatic antioxidant mechanisms such as the production of low-weight antioxidant compounds (i.e., glutathione carotenoids and tocopherol); and (3) stimulate host immunotolerance to mantle tissue antigens (i.e., mycophenolate mofetil) [37]. This aspect is particularly important for evaluating the ability of *P. sterna* to receive not only autografts and allografts but also xenografts as a strategy to improve certain attributes of the resulting pearls [3,4,8]. This research is currently ongoing.

## 5. Conclusions

Evidence from this study indicates that SOD is the more sensitive enzyme for measuring the short-term response of *P. sterna* to relaxation, wounding, and seeding processes during pearl culture. Regardless of the origin of the saibo, it also seems clear that the stress response by the host oyster occurs at all stages of the pearl culturing process, particularly during tissue wounding (gonad and mantle). However, since antioxidant and immune systems are very complex in marine invertebrates and involve different types of effector agents, we recommend studying the effects of seeding surgery over a longer period (up to 72 h) on aspects such as antioxidant and oxidative responses, hematopoiesis (number and content of hemocytes), activity of certain enzymes (i.e., lysozyme, myeloperoxidase, phenoloxidase, and antimicrobial peptides), and the genes encoding their expression. Taken together, this information can broaden the understanding of the physiology associated with seeding surgery in *P. sterna*.

## Data Availability

The data reported in this study will be available for consultation upon request from the corresponding author.
